# Laparoscopic Excision of a Tailgut Cyst With Refractory Pain: A Case Report

**DOI:** 10.7759/cureus.77644

**Published:** 2025-01-18

**Authors:** Masato Kitazawa, Seishu Karasawa, Satoshi Nakamura, Yuta Yamamoto, Yuji Soejima

**Affiliations:** 1 Surgery, Shinshu University School of Medicine, Matsumoto, JPN

**Keywords:** chronic coccygeal pain, laparoscopic excision, minimally invasive surgery, retrorectal cystic hamartoma, tailgut cyst

## Abstract

Tailgut cysts (TGCs) are rare congenital lesions located in the retrorectal space, often remaining asymptomatic and incidentally discovered. However, when symptomatic, they can cause significant discomfort and may require surgical intervention. We report a case of a 40-year-old female who presented with persistent and severe coccygeal pain that significantly impaired her quality of life. Imaging studies revealed a cystic mass consistent with a TGC. Conservative management, including pain control and physical therapy, was ineffective, leading to the decision to surgical treatment. The patient underwent successful laparoscopic excision of the cyst, resulting in complete resolution of symptoms without postoperative complications or recurrence. This case underscores the importance of considering TGCs in the differential diagnosis of chronic coccygeal pain and demonstrates that laparoscopic surgery is a safe and effective treatment option when conservative approaches fail.

## Introduction

Tailgut cysts (TGCs), also known as retrorectal cystic hamartomas, are rare congenital lesions arising from remnants of the embryonic hindgut [1]. These lesions are typically located in the retrorectal space and are histologically classified as dermoid, epidermoid, or enteric cysts. Although often asymptomatic, they may present with nonspecific symptoms such as lower abdominal or perineal pain, constipation, urinary dysfunction, and recurrent infections. Approximately 28% of patients report localized pain in the lower abdomen or perineal area [2]. Moreover, tailgut cysts carry a risk of infection and, albeit rarely, malignant transformation into adenocarcinoma or neuroendocrine tumors [3].

Complete surgical excision is the standard treatment, particularly in symptomatic cases, to prevent recurrence or malignant degeneration. Recently, minimally invasive techniques, such as laparoscopic and robotic-assisted approaches, have gained popularity due to their reduced postoperative morbidity and faster recovery compared to traditional open surgery [4,5].

We present case of a 40-year-old woman with refractory coccygeal pain, in whom a TGC was successfully treated with laparoscopic excision.

## Case presentation

A 40-year-old female was referred to our department from an orthopedic hospital with severe pain localized in the coccygeal region, which was exacerbated by sitting. Her medical history included a uterine polyp that was treated with endometrial curettage. Physical examination revealed significant tenderness in the coccygeal area with a palpable mass on digital rectal examination. Pain was rated 9/10 on the numeric rating scale (NRS).

Imaging studies

Computed Tomography (CT) Scan

**Figure 1:**
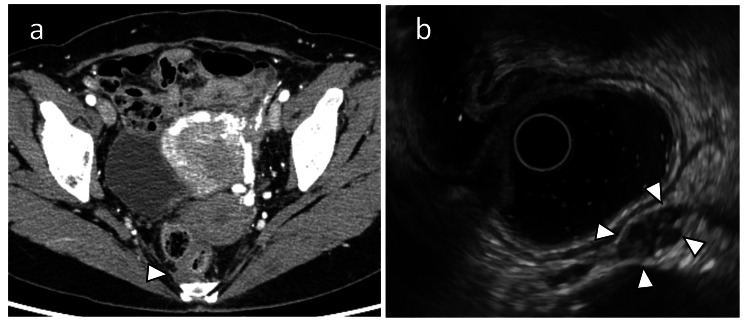
Preoperative CT and transrectal ultrasonography (a) Contrast-enhanced CT scan revealing a poorly defined, low-density mass with no contrast enhancement anterior to the coccyx (arrowhead). (b) Transrectal endoscopic ultrasound showing a 12 mm hypoechoic mass with internal heterogeneity (arrowheads). A biopsy was performed under endoscopic ultrasound guidance; however, no neoplastic lesions were identified, making the diagnosis challenging. CT: computed tomography

A low-density cystic mass was detected anterior to the coccyx, without enhancement, after contrast administration (Figure 1a).

Endoscopic Ultrasound

A 12-mm cystic lesion with heterogeneous echogenicity was observed. Biopsy results were inconclusive and failed to reveal any neoplastic changes (Figure 1b).

Magnetic Resonance Imaging (MRI)

**Figure 2:**
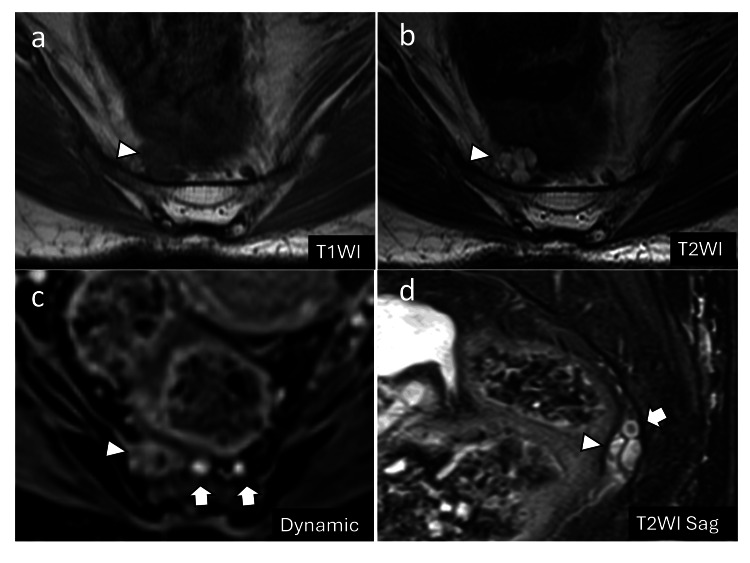
Preoperative MRI (a, b) MRI showing a multilocular cystic mass with low signal intensity on T1-weighted images and high signal intensity on T2-weighted images (arrowheads). (c, d) Contrast-enhanced MRI revealing the median sacral artery and vein (arrows) located adjacent to the mass (arrowheads). MRI: magnetic resonance imaging

T1-weighted images revealed a low signal intensity, while T2-weighted images showed a high signal intensity, consistent with a multiloculated cystic lesion (Figures 2a, 2b). The mass was closely associated with the median sacral vein, raising concerns about the potential bleeding risks during surgery (Figures 2c, 2d).

Surgical intervention

The patient initially underwent conservative management, which included nerve block injections and oral analgesics (acetaminophen, non-steroidal anti-inflammatory drugs, and pregabalin), but there was little symptom improvement. Due to persistent pain (NRS score of 7) one year after diagnosis, surgical excision became necessary.

**Figure 3:**
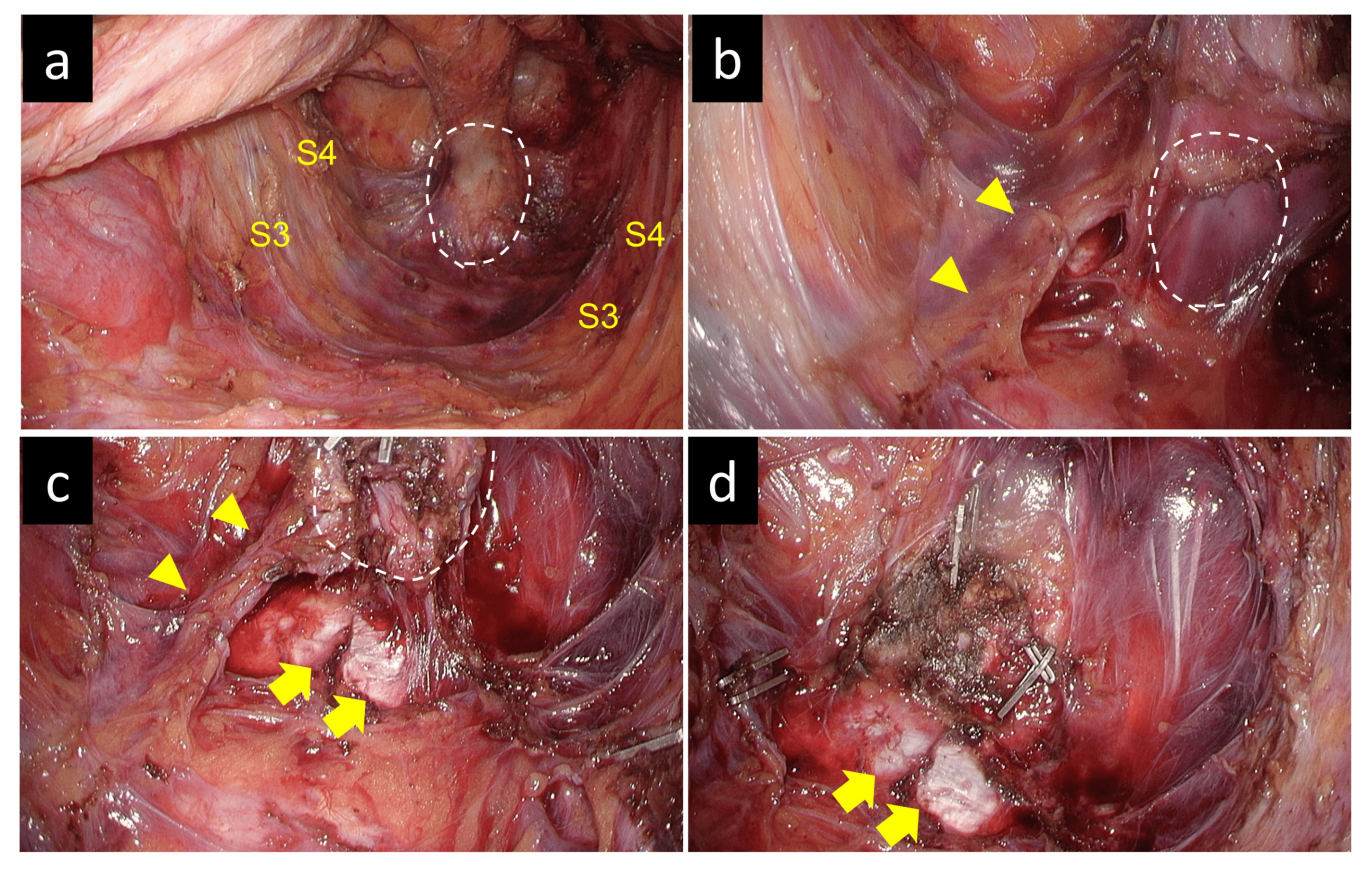
Intraoperative findings (a) The S3 and S4 nerves were preserved, and dissection was performed in the retrorectal space, with extensive dissection of the supralevator space. The mass was located on the ventral side of the coccyx, within the area outlined by the dotted line. (b) The median sacral artery and vein were visible and located close to the mass (outlined by the dotted line). (c) The periosteum of the coccyx was dissected, and the mass (outlined by the dotted line) was dissected dorsally. The median sacral artery and vein adjacent to the mass were clipped and divided. (d) After tumor resection, the coccyx was exposed, and the median sacral artery and vein and draining veins adjacent to the mass were clipped, ensuring no bleeding during mass removal.

The surgery lasted three hours and 24 min with minimal blood loss. Given that robotic surgery is considered useful but is not currently covered by insurance in Japan, a laparoscopic approach was utilized for this procedure. The cyst was successfully dissected and removed while preserving critical structures, including the sacral nerves (S3 and S4). The space above the levator ani muscle was dissected to ensure adequate visualization (Figure 3a). The median sacral artery and vein, which were in close proximity to the cyst, required clip ligation (Figures 3b, 3c). The cyst was then carefully dissected from the sacral and coccygeal periosteum, and complete excision was achieved (Figure 3d).

Postoperative course

The patient recovered uneventfully and was discharged on postoperative day 7. All pain medications were discontinued on postoperative day 14. The only complication was transient constipation (Clavien-Dindo Grade 2). Follow-up imaging confirmed the complete removal of the cyst, and the patient remained symptom-free.

Histopathological findings

**Figure 4:**
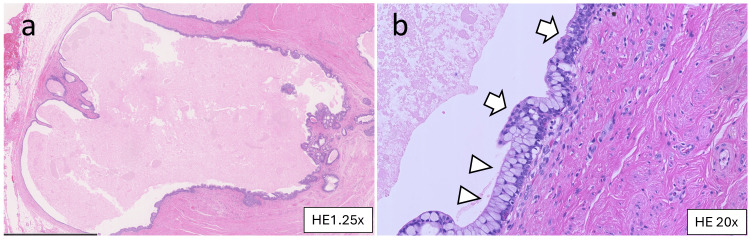
Histopathological findings (a, b) Hematoxylin and eosin staining. The cystic lesion is lined with multilayered columnar (arrowheads) and transitional (arrows) epithelium. There is connective tissue and smooth muscle tissue around the cyst.

Pathological examination confirmed the diagnosis of a TGC, characterized by a cystic lesion lined with columnar epithelium and surrounding smooth muscle. No malignant transformations were observed (Figures 4a, 4b).

## Discussion

TGCs are rare congenital lesions that present a diagnostic challenge, with a reported malignancy potential of 8-14% [6,7]. Complete surgical excision remains the recommended treatment to prevent recurrence or malignant transformation [7,8]. The surgical approach to TGCs typically depends on their size, location, and proximity to critical structures [9]. Both transabdominal and transsacral approaches are viable options. In the present case, the cyst was located close to the midline sacral vessels, making precise control of the vasculature crucial. The laparoscopic approach provided excellent visualization, allowing us to safely navigate around the sacral venous plexus and perform complete resection while minimizing the risk of injury to adjacent structures and excessive bleeding.

Another critical consideration is that TGCs may be associated with chronic pain due to compression of surrounding tissues, as in our case. In particular, the coccygeal region is richly innervated by the sensory branches of the sacral plexus, including the S4 and S5 nerves, which have the potential to cause significant pain when irritated by local structures such as TGCs. This type of neuropathic pain, known as coccygodynia, has been noted in the literature as a possible consequence of such irritation [10-12]. Although the exact severity of pain varies, a single institution report indicated that 29% of patients with TGCs experienced lower abdominal or perineal pain [2]. Additionally, case reports have documented patients with significant pain associated with these cysts, highlighting the potential impact on quality of life in such cases [13].

In this particular case, the patient had been suffering from coccygeal pain for several years despite various treatments such as oral medications and nerve blocks. The patient sought care from multiple medical institutions without any significant relief. Following surgical excision of the TGC, the patient experienced complete relief from chronic pain and no longer required pain medications. This highlights the potential of surgical intervention not only for removing the cyst but also for resolving associated symptoms, such as chronic pain, that are resistant to conservative treatments.

## Conclusions

Laparoscopic excision of TGCs is a safe and effective treatment option for symptomatic patients, offering the advantages of minimal invasiveness, reduced postoperative pain, and faster recovery. This case highlights the critical role of thorough preoperative imaging and meticulous surgical planning in identifying anatomical complexities and minimizing intraoperative complications. Complete resection of TGCs is essential due to the potential risk of malignant transformation, underscoring the need for precise surgical techniques to achieve clear margins. This approach not only ensures symptom relief but also reduces the likelihood of recurrence, emphasizing laparoscopic surgery as a reliable standard treatment.
